# Evaluation of the middle cerebral artery occlusion techniques in the rat by in-vitro 3-dimensional micro- and nano computed tomography

**DOI:** 10.1186/1471-2377-10-36

**Published:** 2010-05-28

**Authors:** Alexander C Langheinrich, Mesut Yeniguen, Anne Ostendorf, Simone Marhoffer, Marian Kampschulte, Georg Bachmann, Erwin Stolz, Tibo Gerriets

**Affiliations:** 1 Department of Radiology, Justus-Liebig University Giessen, Giessen, Germany; 2 Department of Neurology, Experimental Neurology Research Group, Justus-Liebig University Giessen, Giessen, Germany; 3 Department of Radiology, Kerckhoff Clinic, Bad Nauheim, Germany

## Abstract

**Background:**

Animal models of focal cerebral ischemia are widely used in stroke research. The purpose of our study was to evaluate and compare the cerebral macro- and microvascular architecture of rats in two different models of permanent middle cerebral artery occlusion using an innovative quantitative micro- and nano-CT imaging technique.

**Methods:**

4h of middle cerebral artery occlusion was performed in rats using the macrosphere method or the suture technique. After contrast perfusion, brains were isolated and scanned en-bloc using micro-CT (8 μm)^3 ^or nano-CT at 500 nm^3 ^voxel size to generate 3D images of the cerebral vasculature. The arterial vascular volume fraction and gray scale attenuation was determined and the significance of differences in measurements was tested with analysis of variance [ANOVA].

**Results:**

Micro-CT provided quantitative information on vascular morphology. Micro- and nano-CT proved to visualize and differentiate vascular occlusion territories performed in both models of cerebral ischemia. The suture technique leads to a remarkable decrease in the intravascular volume fraction of the middle cerebral artery perfusion territory. Blocking the medial cerebral artery with macrospheres, the vascular volume fraction of the involved hemisphere decreased significantly (p < 0.001), independently of the number of macrospheres, and was comparable to the suture method. We established gray scale measurements by which focal cerebral ischemia could be radiographically categorized (p < 0.001). Nano-CT imaging demonstrates collateral perfusion related to different occluded vessel territories after macrosphere perfusion.

**Conclusion:**

Micro- and Nano-CT imaging is feasible for analysis and differentiation of different models of focal cerebral ischemia in rats.

## Background

Over the last decade, the rat has become the predominant species for models of focal cerebral ischemia. Up to now, different techniques simulating human cerebral ischemia have been established in rats. Among the endovascular techniques for middle cerebral artery occlusion (MCAO), the suture occlusion technique in rats is the most frequently used method [1-3]. In this model, a monofilament is implemented into the internal carotid artery (ICA) until it blocks blood flow to the middle cerebral artery (MCA). This technique provides reproducible MCA territory infarctions and allows reperfusion by releasing the suture. Permanent MCAO with the suture technique, however, has one disadvantage: insertion of the suture occludes the entire course of the ICA, including the hypothalamic artery. This approach leads to hypothalamic infarction that cause severe hyperthermia with effects on infarct growth, confounding treatment effects [4,5].

The recently introduced macrosphere model has been developed to overcome these side effects by the intra-arterial embolization of TiO_2 _spheres which selectively block blood flow to the MCA main stem without obstructing the hypothalamic artery [4,6]. This model therefore avoids hypothalamic infarction and pathological hyperthermia in permanent MCA occlusion.

Reliable visualization and detection of cerebral ischemia and particularly the underlying vascular pathology remain a major challenge in vascular research. However, detailed anatomical data about the vascular status, are generally sparse because (i) of the difficulty in their quantification, (ii) of difficulty to demonstrate their interconnectivity to other vessels (collaterals), (iii) of their very fine anatomy in animal models of focal cerebral ischemia.

In the last decade, micro-computed tomography (micro-CT) made it possible to overcome many of these difficulties and permits analysis of the complete 3D branching architecture of small vessels in microscopic detail. Previously, we reported micro-CT's technical feasibility to visualize the intacerebral arteries [7] in apoE^-/-^LDL^-/- ^double knockout mice. Hence, the purpose of our study was to evaluate micro- and nano-CT's technical feasibility to detect and differentiate structural and functional vascular alterations in two different models of permanent arterial cerebral ischemia in rats.

## Methods

### Animal Preparation

All procedures were performed in accordance with our institutional guidelines and the German animal protection legislation. Male Sprague-Dawley rats (277 to 338 g bodyweight; Harlan Winkelmann, Borchen, Germany) were anesthetized with 5% isoflurane delivered in air for 2 minutes. Anesthesia was maintained with 2% to 3% isoflurane delivered in air at 0.5 L/min during surgery. Body temperature was continuously monitored with a rectal probe and maintained at 36.5°C to 37.0°C. The right external carotid artery (ECA) was ligated and transsected to create an ECA stump with a length of ~5 mm. The pterygopalatine branch of the ICA was also occluded.

### Suture Model

A 4-0 silicone-coated nylon suture was introduced through the ECA stump in animals (n = 10) as described previously [4]. The occluder was advanced into the ICA 16 to 18 mm beyond the carotid bifurcation until mild resistance indicated that the tip was lodged in the anterior cerebral artery and thus blocked blood flow to the MCA.

### Macrosphere Model

PE-50 tubing, filled with saline and 4 TiO2 macrospheres (diameter, 0.315 to 0.355 mm; BRACE GmbH, Alzenau, Germany), was inserted into the ECA stump (n = 8). The tip of the tubing was placed in the carotid bifurcation without affecting the blood flow to the ICA. Then the macrospheres were advanced separately into the ICA by a slow injection of 0.05 mL saline each, until they were moved passively into the cerebral circulation by the physiological blood flow [6].

### Control Animals

Four additional rats were not subjected to MCA occlusion and served as controls.

### Post mortem preparation

After 4h, the animals were anesthetized with 5% isoflurane delivered in air for 2 minutes. Anesthesia was maintained with 2% to 3% isoflurane delivered in air at 0.5 L/min during surgery.

After thoracotomy, PE-50 tubing was inserted into the left ventricle, followed by incision of the right atrium. Then the animal's circulation was flushed with heparinized saline.

The ascending and descending aorta, the subclavian arteries, the external carotid arteries and the pterygopalatine arteries were ligated to avoid unnecessary contrast filling. Then 5 ml Microfil^® ^(Flow Tech, Inc., Carver, Massachusetts, USA) was prepared according to the manufacturer's instructions and injected through the aortic arch to fill the arterial and venous cerebral circulation via the internal carotid and the vertebral arteries. Excessive dilatation of the aorta during the injection process was avoided in order to ensure physiological perfusion pressures. After 45 minutes, Microfil formed an elastomeric gel at room temperature. Then the brains including the intact dura mater were removed from the skull and immersed in 4.5% formalin. Contrast perfusion was performed after 4 hours. Localisation of the suture and the macrospheres within the basal cerebral arteries as well as the presence of subarachnoidal blood was assessed by visual inspection and carefully documented.

According to the previously published criteria, sufficient occlusion of the middle cerebral artery was assumed, if the origin of the MCA-mainstem or all arteries providing blood to the MCA mainstem (i.e. anterior cerebral artery and internal carotid artery) were blocked by macrospheres. Subarachnoid hemorrhage led to exclusion from the study [6].

### Micro-Computed Tomography

All samples were scanned in a micro-computed tomograph (micro-CT) manufactured and developed by SkyScan (SkyScan1072_80 kV; Belgium). The X-ray system is based on a microfocus tube (20-80 kVp, 0-100 μA) reaching a minimum spot size of 8 μm at 8W generating projection images irradiating X-rays in cone-beam geometry. This system has been described in detail before [8,9]. The resulting 3D images were displayed using image analysis software (Analyze^® ^8.0; Biomedical Imaging Resource, Mayo Clinic, Rochester, MN). For this study, our micro-CT scanner was configured so that the side dimension of the cubic voxels was 12 μm. Gray scale attenuations of micro-CT images were measured in the right and left hemisphere in controls and animals after middle artery occlusion (n = 80/brain) using the ANALYZE^© ^software package. Therefore, non-contrast perfused animals were used in the Suture Model and in the control group (each n = 3). For determination of relative attenuation values, rectangular regions of interest (ROI; side length, 0.5 mm at 8 bit) were established manually in areas by non-visible assessment of gray-scale differences.

### Nano-Computed Tomography

For more detailed analysis of the brain microvessels, samples were cut with a side length of 4 mm and rescanned using a nano-computed tomograph (Nano-CT_2011), manufactured and developed by SkyScan^® ^(Kontich, Belgium). The microfocus X-ray source is a pumped type source (open type x-ray source) with a LaB_6 _cathode. The electron beam is focused by two electromagnetic lenses onto the surface of an x-ray target. The x-ray target (Au) contains a thin tungsten film plated on the surface of the beryllium window producing x-ray emission reaching a minimum spot size of < 400 nm. At this small spot size, small-angle scattering enhances object details down to 150 nm isotropic voxels size. The X-ray detector consists of a 12-bit digital, water-cooled CCD high-resolution (1280 × 1024 pixel) camera with fibre optic 3.7:1 coupling to an X-ray scintillator and digital frame-grabber. In our experimental setting, samples were positioned on a computer controlled rotation stage and scanned 180° around the vertical axis in rotation steps of 0.25 degrees at 40 kVp. Acquisition time for each view was 2.4 seconds. Relative position of the object to the source determines geometric magnification and thus the pixel size defined by the cone-beam geometry of the system. Maximum possible magnification is also limited by the specimen size, which has to be within the cone-beam in its horizontal diameter. Raw data were reconstructed with a modified Feldkamp cone-beam reconstruction modus resulting in two dimensional 8-bit gray-scale images consisting of cubic voxels.

### Statistical Analysis

Statistical analysis was performed using JMP 6.0 (SAS Institute, Cary, NC, USA). All data in the text and figures are presented as mean ± SEM. Vascular volume fraction and gray scale attenuation differences were analyzed using unpaired t test and one-way ANOVA. A value of p < 0.05 was considered significant in all analyses.

## Results

### General Consideration

Micro-CT and Nano-CT imaging did not provoke any radiographic artefacts, interfering with image analysis. Using micro-CT imaging, a continuous visualization and quantification of the intracerebral vasculature can be performed. For more detailed information on cerebral microvessels, high-resolution nano-CT imaging can also be used to analyse distinct regions of interest more accurately.

### Suture Model

**Figure 1:**
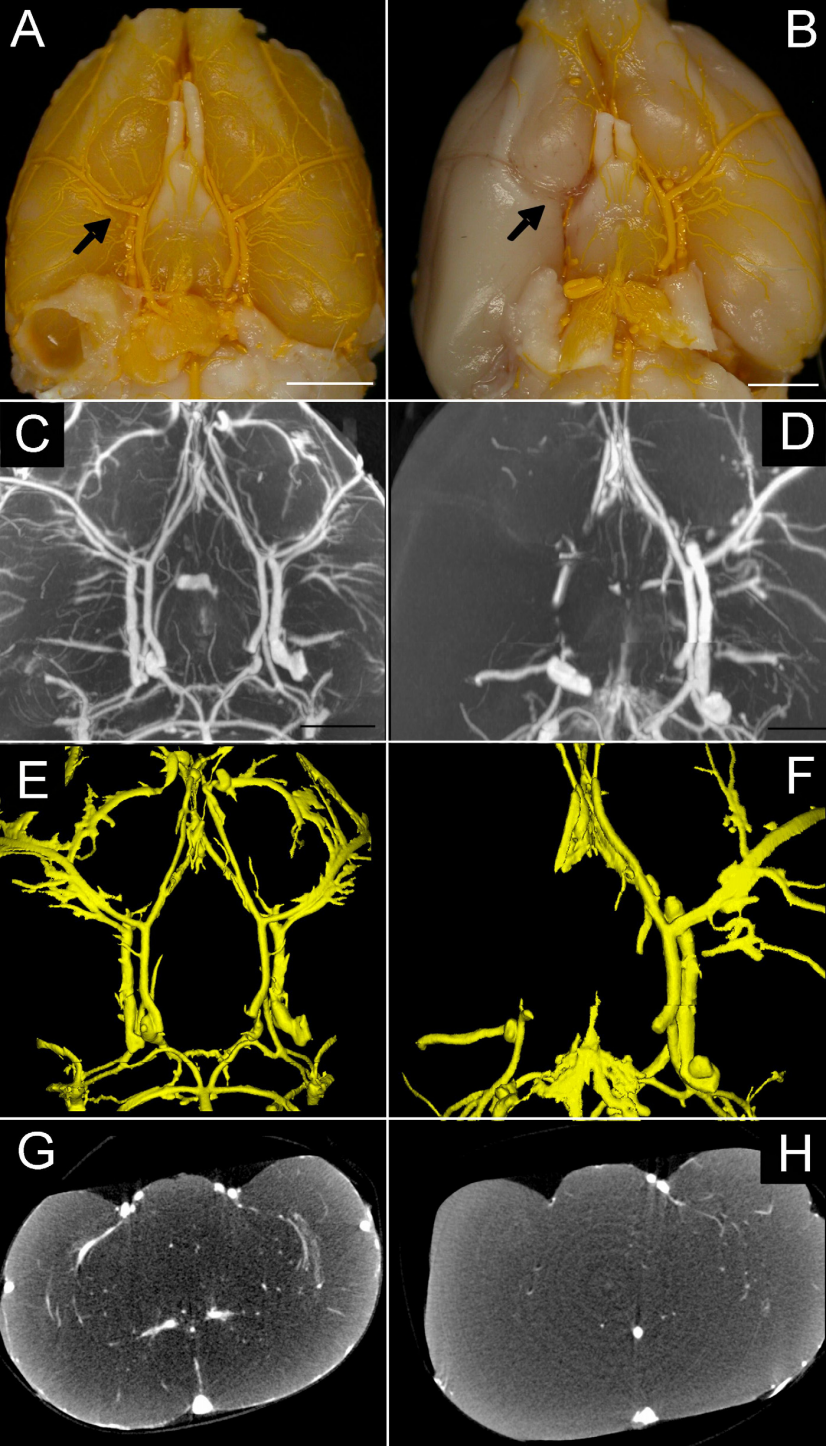
**Macroscopic photograph of the rat brain from controls (A) and the suture model (B, bar = 4 mm)**. The suture has been removed and after contrast perfusion, the occluded right part of the circle of willis and the branching middle cerebral artery are demonstrated. Maximum intensity projection (C, D), surface-rendering (E, F) and coronar single-slice micro-CT demonstrate the occluded middle cerebral artery (D, F, H) compared to controls (C, E, G).

Figure 1 shows micro-CT findings of a control animal (left column) and of a rat subjected to suture-MCA occlusion (right column). Subarachnoid hemorrhage was found in one of 10 animals as a result of perforation of the intracranial portion of the anterior cerebral artery. This animal was excluded from further evaluation.

**Figure 2:**
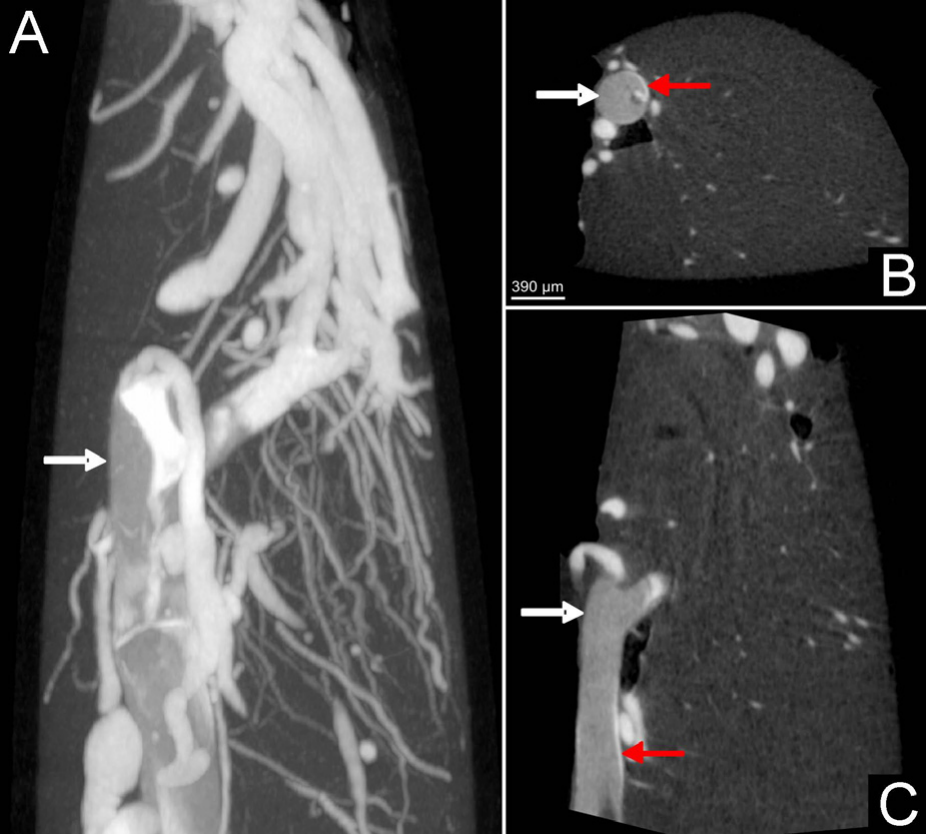
**Contrast agent at the top of the suture (white arrow) indicating partial perfusion in the anterior cerebral artery as demonstrated with nano-CT imaging**. Note the sparse contrast perfusion between the suture and the vascular wall (red arrow) indicating the non-complete occlusion of the vessel.

Permanent occlusion resulted in a significantly lower vascular volume fraction as compared to the non-occluded hemisphere (4.02 ± 1.67 mm^3 ^vs. 11.5 ± 1.05 mm^3^; p < 0.001; Table 1). Nano-CT imaging indicates a faint contrast filling of the vasculature distal to the occlusion derived by crossflow-filling from the contralateral hemisphere. Moreover, a sparse portion of dye was visible between the suture and the vessel wall, indicating a non-complete occlusion of the internal carotid artery by the suture (Figure 2).

**Table 1:** Macrospheres versus Suture Technique.

		Number of Macrospheres	Vessel Diameter (μm)	Macrosphere Diameter (μm)	Occluded Vessel Territories	Collaterals via	Vascular Volume (mm^3^)
**Macrospheres**	**Anterior Cerebral Artery**	7	186 ± 6*	334 ± 11	No	Contralateral Hemisphere	10.9 ± 0.9
	**Middle Cerebral Artery**	7	203 ± 13	335 ± 7	Partial	Thalamo-striatal Arteries	5.5 ± 1.3*
	**Posterior Cerebral Artery**	5	175 ± 11*	333 ± 4	No	Contralateral Hemisphere	11.4 ± 0.9
	**Circle of Willis**	16	205 ± 16	333 ± 8	Partial	Unknown	8.2 ± 0.8
							

**Suture**	**Occluded Hemisphere**				Partial	Unknown	4.02 ± 1.6*
	**Non-Occluded Hemisphere**				No		11.5 ± 1.1
						

**Controls**							11.6 ± 1.2

The vessel diameter shows significant differences between the anterior and posterior versus the middle cerebral artery and the circle of Willis (* p < 0.002). The macrosphere diameter is not related to the occluded vessel diameter (p < 0.2) but the diameter of the macrospheres shows significantly higher values than the occluded vessel (p < 0.001). Macrospheres in the middle cerebral artery showing the same vascular volume fraction as the suture technique.

Gray scale densities were measured and matched to the middle cerebral artery perfusion territories. Animals showed significantly lower gray scale attenuation values as a result of progressive edema.

### Macrosphere Model

**Figure 3:**
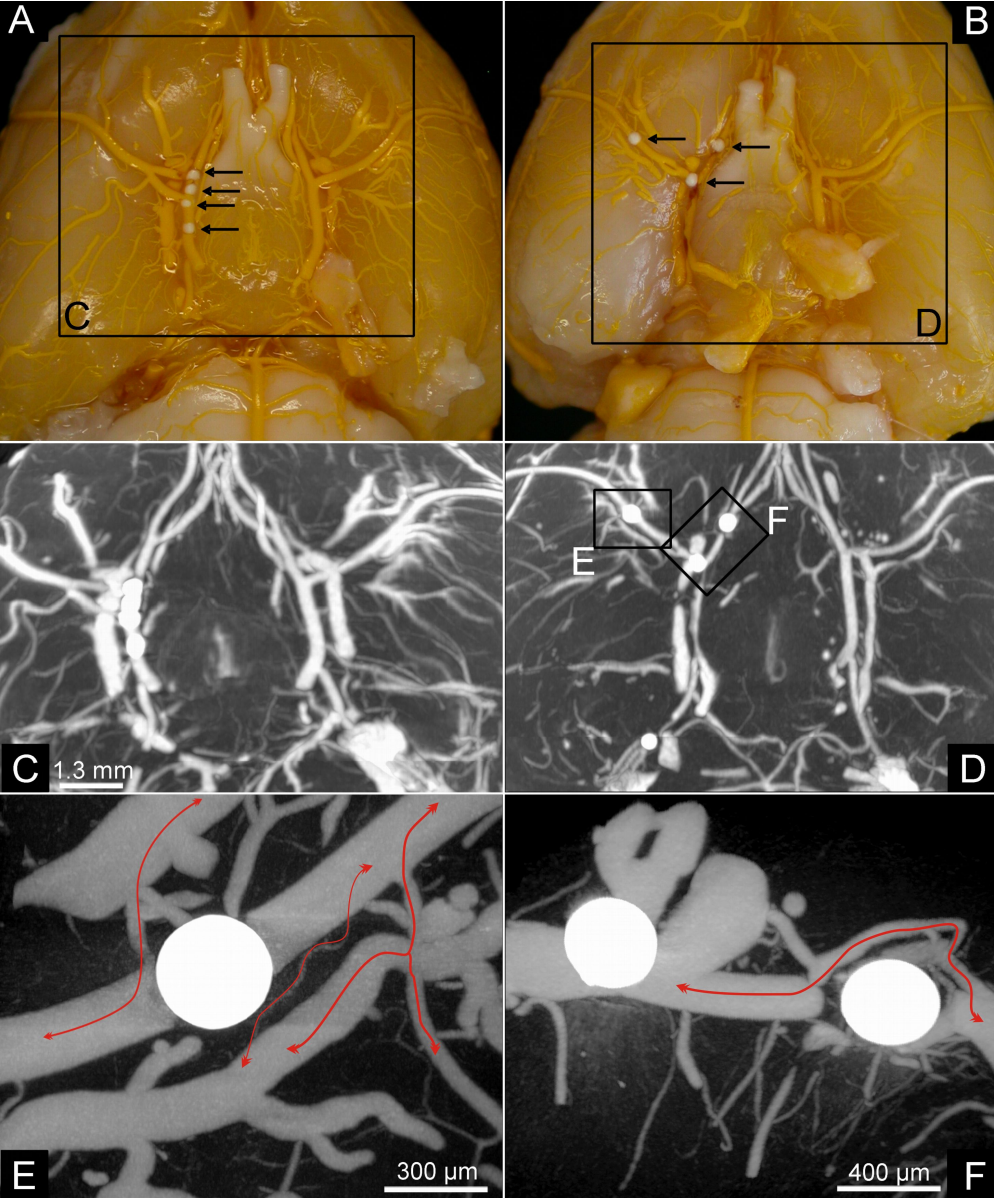
**Macrosphere Model**. Macroscopic image (A, B) of macrospheres present in the circle of Willis (A) and in the circle of Willis, the anterior cerebral artery and the middle cerebral artery (B). Micro-CT maximum intensity projection (C, D) also demonstrate the macrosphere perfusion territories. Nano-CT imaging (E, F) demonstrate collateral perfusion with contrast-enhanced afferent and efferent vessel perfusion.

Figures 3 demonstrate the typical distribution of macrospheres within the basal cerebral arteries. All administered spheres were detected with micro- and nano-CT imaging.

**Figure 4:**
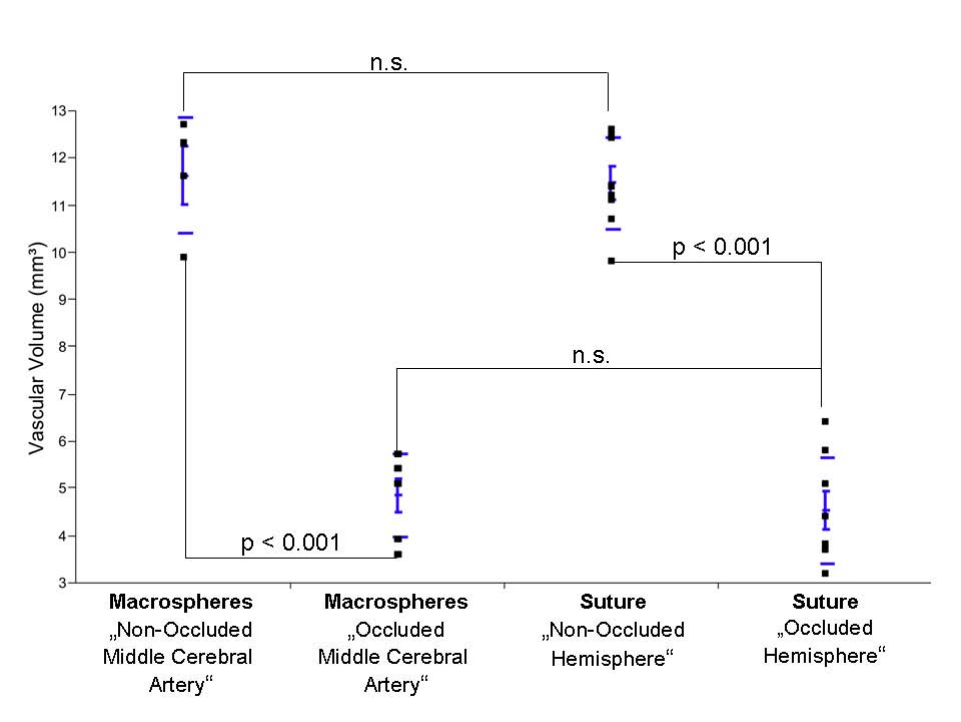
**Vascular volume fraction (VVF) in Controls, the Macrosphere- and the Suture Model**. Significant differences were found between controls and the macrosphere blocked media territory and the suture technique (p < 0.001). Macrospheres in the middle cerebral artery obtained the same results as the suture technique (p < 0.2).

In 2 animals the macrospheres were lodged exclusively in the anterior and/or posterior cerebral artery without blocking blood flow to the MCA. These animals therefore were excluded from the study since they did not fulfil the predefined criteria for MCA occlusion. As expected, these rats did not show significant differences in the total vascular volume fraction, as compared to controls (p < 0.2). In contrast, animals with correct macrosphere localisation demonstrate a significant decrease (p < 0.001) in the vascular volume fraction (5.5 ± 1.3 mm^3^) compared to controls/non-occluded hemisphere (11.6 ± 1.2 mm^3^), indicating sufficient MCA occlusion (Figure 4 and Table 1).

**Figure 5:**
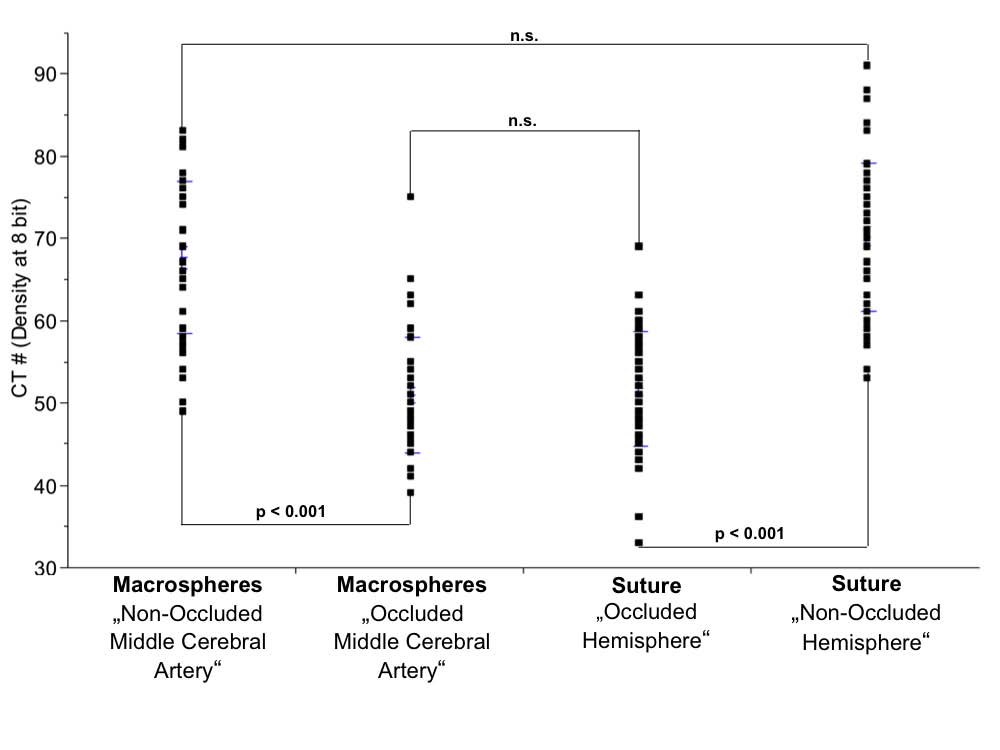
**Gray scale attenuation differences obtained in Controls, the Macrosphere- and Suture Model**. Significant differences in CT gray scale attenuations were obtained in different perfusion territories between the macrospheres and the suture technique. No significant differences were obtained for macrospheres in the middle cerebral artery and the suture model.

Since the diameter of the macrospheres (334 ± 10 μm) is significantly higher than the occluded vessel (175 to 205 μm), a complete occlusion can be assumed. However, micro and nano-CT indicates contrast filling of the vasculature distal to the occlusion site (Figure 1). High-resolution nano-CT imaging indicates that distal filling of the middle cerebral artery results from collaterals in the vicinity of the occlusion site (Figure 1E+F, black curved arrows). No contrast agent was detectable between the macrospheres and the vessel wall (Figure 1E+F). As a concomitant effect, gray scale value attenuations in the same vascular perfusion territories also significantly decreased (Figure 5) indicating progressive edema due to focal ischemia. No case of subarachnoid hemorrhage was found in the macrosphere model.

## Discussion

For several decades rodent cerebral ischemia models represent a corner stone in stroke research and provided important insights into the underlying pathophysiology of ischemic stroke. Particularly the suture technique has been used frequently, because it is highly reliable and it allows simulating both fundamental conditions of cerebral ischemia: *permanent *vessel occlusion and *ischemia followed by reperfusion*. Both conditions are relevant to human stroke and should be taken into account i.e. for the preclinical testing of neuroprotective drugs [10]. Since the pathophysiology of "permanent" and "transient" focal cerebral ischemia is essentially different, animal models should reliably provide these conditions. Permanent MCA occlusion with use of the suture model, however, has been come under criticism for frequent inadvertent premature reperfusion, as documented by laser Doppler measurements - a side-effect that potentially undermines the strict differentiation between permanent and transient ischemia [3]. The macrosphere-technique in contrast, due to the very tight occlusion of the basal cerebral arteries, has the potential to overcome this side-effect by avoiding bypass blood flow between the vessel wall and the occluder.

The present paper evaluated and compared the vascular anatomy of the permanent suture and the macrosphere MCA occlusion method by using a novel high resolution imaging technique.

The present data indicate that both models of permanent vessel occlusion do not completely block blood flow to the MCA territory. This finding is in accordance with many previous observations using laser Doppler monitoring during the occlusion process with the suture technique, were blood flow in the MCA territory does not drop to zero but is typically reduced by 70 to 90% [3].

With the use of micro- and nano-CT, we now could visualize for the first time the morphological basis of the remaining perfusion in the two permanent MCA-occlusion models.

1. *Distant collateral blood flow *from the contralateral hemisphere (cross-flow) was present in both occlusion models, most likely via connections between the ipsilateral and contralateral anterior cerebral artery.

2. *Local collateral blood flow *in the vicinity of the occluder was an unexpected finding in the macrosphere-model (Figure 1E+F) as well as in the suture technique (Figure 3). Particularly in the macrosphere occlusion method small collateral vessels shifted blood over a short distance from proximal to distal segment of the occluded MCA. This phenomenon was detectable to a much lesser extent in the suture technique, since the suture blocks the internal carotid artery over its entire distance, which impedes short distance collateral flow.

3. *Inadvertent bypass-perfusion *between the occluder and the occluded vessel could be detected in the suture model (Figure 3). This finding is most likely related to a discrepancy between the suture diameter and the vessel caliber. Schmidt-Elsaesser and coworkers [3] found a higher proportion of inappropriate MCA occlusion when smaller sutures are used and Laing et al. [11] stated that silicone coated (and therefore thicker) filaments provide denser ischemia than uncoated sutures, would support this hypothesis. The use of thicker sutures than coated 4-0 filaments, however, seems not to be feasible, since they are difficult to insert through the base of the skull. Inadvertent bypass-perfusion was not detected in the macrosphere model.

In normal Wistar rats, extensive anastomosis among the terminal branches of the three large cerebral arteries have been described and is highest between the anterior and middle cerebral arteries, and also between between the middle and posterior cerebral arteries [12-14]. Micro- and nano-CT imaging demonstrates that collateral supply to the cortical vascular beds protects in part against complete vascular occlusion and is strongly related to the blocked macrosphere perfusion territory. Those findings are in line with the published literature [15-17], indicating pre-existing anastomosis and collaterals after middle cerebral artery occlusion in rats.

Imaging methods are indispensable for studying the vessel architecture in animal models of vascular disorders. Despite the power and utility of these research methods, there is still a large difference in spatial resolution between microscopy-based imaging methods and clinical methods. High-resolution magnetic resonance angiography (MRA) has been introduced as a means to investigate the arterial cerebrovascular hemodynamics noninvasively in rats [1,18] and mice [19]. Up to now, MRI imaging is the most frequently used method to investigate cerebral perfusion in experimental [20,21] and clinical conditions [22,23]. Major limitations using MRI imaging is the spatial resolution needed to visualize and quantify changes in the microvasculature. Clinical and experimental studies addressed these aspects using 3, 4.7, 7 and 11.7 Tesla systems [1,24-27].

Micro-CT has made it possible to largely overcome these difficulties and permit analysis of the complete 3D branching architecture of small vessels in microscopic detail [28,29]. Moreover, Micro-CT imaging makes it possible to quantify the number and spacing of blood vessels, [30] and vascular permeability [31]. Micro-CT in the present study emerged as a sensitive technique to detect vessel alterations in different models of focal brain ischemia. The 3D micro-CT images have several advantages over 2D histology, as one can unambiguously identify a vessel along its entire length from the vessel's origin to the terminal region.

## Conclusion

This study demonstrates a methodological approach to preserve and examine the rat cerebral vasculature under physiological and pathological conditions. To our knowledge, this is the first study using micro-CT and nano-CT technique for imaging the intracerebral vasculature non-destructively in different models of focal brain ischemia.

The present data indicate that rodent models of "permanent" MCA occlusion provide a certain amount of remaining perfusion via different collateral pathways and (in case of the suture technique) also by inadvertent bypass-perfusion along the filament. These findings emphasize the thesis, that unadulterated permanent ischemic conditions can not be simulated with the present rodent stroke models since they all contain some elements of reperfusion.

## Competing interests

The authors declare that they have no competing interests.

## Authors' contributions

ACL, MK, and AO carried out the micro-CT studies and drafted the manuscript. Animal preparation and perfusion was performed by MY and SM. GB, ES and TG participated in its design and coordination and helped to draft the manuscript. All authors read and approved the final manuscript.

## Pre-publication history

The pre-publication history for this paper can be accessed here:

http://www.biomedcentral.com/1471-2377/10/36/prepub

## References

[B1] Gerriets T Stolz E Walberer M Muller C Rottger C Kluge A Kaps M Fisher M Bachmann G Complications and pitfalls in rat stroke models for middle cerebral artery occlusion: a comparison between the suture and the macrosphere model using magnetic resonance angiography Stroke 2004 35 2372 2377 10.1161/01.STR.0000142134.37512.a7 15345802

[B2] Gerriets T Stolz E Walberer M Muller C Kluge A Kaps M Fisher M Bachmann G Middle cerebral artery occlusion during MR-imaging: investigation of the hyperacute phase of stroke using a new in-bore occlusion model in rats Brain Res Brain Res Protoc 2004 12 137 143 10.1016/j.brainresprot.2003.08.006 15013464

[B3] Schmid-Elsaesser R Zausinger S Hungerhuber E Baethmann A Reulen HJ A critical reevaluation of the intraluminal thread model of focal cerebral ischemia: evidence of inadvertent premature reperfusion and subarachnoid hemorrhage in rats by laser-Doppler flowmetry Stroke 1998 29 2162 2170 9756599 10.1161/01.str.29.10.2162

[B4] Gerriets T Stolz E Walberer M Kaps M Bachmann G Fisher M Neuroprotective effects of MK-801 in different rat stroke models for permanent middle cerebral artery occlusion: adverse effects of hypothalamic damage and strategies for its avoidance Stroke 2003 34 2234 2239 10.1161/01.STR.0000087171.34637.A9 12920258

[B5] Memezawa H Zhao Q Smith ML Siesjo BK Hyperthermia nullifies the ameliorating effect of dizocilpine maleate (MK-801) in focal cerebral ischemia Brain Res 1995 670 48 52 10.1016/0006-8993(94)01251-C 7719723

[B6] Gerriets T Li F Silva MD Meng X Brevard M Sotak CH Fisher M The macrosphere model: evaluation of a new stroke model for permanent middle cerebral artery occlusion in rats J Neurosci Methods 2003 122 201 211 10.1016/S0165-0270(02)00322-9 12573479

[B7] Langheinrich AC Michniewicz A Bohle RM Ritman EL Vasa vasorum neovascularization and lesion distribution among different vascular beds in ApoE-/-/LDL-/- double knockout mice Atherosclerosis 2007 191 73 81 10.1016/j.atherosclerosis.2006.05.021 16806224

[B8] Langheinrich AC Leithauser B Greschus S Von Gerlach S Breithecker A Matthias FR Rau WS Bohle RM Acute rat lung injury: feasibility of assessment with micro-CT Radiology 2004 233 165 171 10.1148/radiol.2331031340 15317950

[B9] Langheinrich AC Bohle RM Breithecker A Lommel D Rau WS [Micro-computed tomography of the vasculature in parenchymal organs and lung alveoli] Rofo 2004 176 1219 1225 15346254 10.1055/s-2004-813403

[B10] Recommendations for standards regarding preclinical neuroprotective and restorative drug development Stroke 1999 30 2752 2758 10583007 10.1161/01.str.30.12.2752

[B11] Laing RJ Jakubowski J Laing RW Middle cerebral artery occlusion without craniectomy in rats. Which method works best? Stroke 1993 24 294 298 8421831 10.1161/01.str.24.2.294

[B12] Coyle P Jokelainen PT Dorsal cerebral arterial collaterals of the rat Anat Rec 1982 203 397 404 10.1002/ar.1092030309 7137595

[B13] Coyle P Dorsal cerebral collaterals of stroke-prone spontaneously hypertensive rats (SHRSP) and Wistar Kyoto rats (WKY) Anat Rec 1987 218 40 44 10.1002/ar.1092180108 3605659

[B14] Coyle P Cerebral collateral patterns in normal Wistar rats Acta Physiol Scand Suppl 1986 552 5 8 3468749

[B15] Coyle P Middle cerebral artery occlusion in the young rat Stroke 1982 13 855 859 7147305 10.1161/01.str.13.6.855

[B16] Coyle P Spatial relations of dorsal anastomoses and lesion border after middle cerebral artery occlusion Stroke 1987 18 1133 1140 3686589 10.1161/01.str.18.6.1133

[B17] Coyle P Heistad DD Blood flow through cerebral collateral vessels one month after middle cerebral artery occlusion Stroke 1987 18 407 411 3564097 10.1161/01.str.18.2.407

[B18] Reese T Bochelen D Sauter A Beckmann N Rudin M Magnetic resonance angiography of the rat cerebrovascular system without the use of contrast agents NMR Biomed 1999 12 189 196 10.1002/(SICI)1099-1492(199906)12:4<189::AID-NBM557>3.0.CO;2-O 10421910

[B19] Beckmann N High resolution magnetic resonance angiography non-invasively reveals mouse strain differences in the cerebrovascular anatomy in vivo Magn Reson Med 2000 44 252 258 10.1002/1522-2594(200008)44:2<252::AID-MRM12>3.0.CO;2-G 10918324

[B20] Rother J Waggie K van Bruggen N de Crespigny AJ Moseley ME Experimental cerebral venous thrombosis: evaluation using magnetic resonance imaging J Cereb Blood Flow Metab 1996 16 1353 1361 10.1097/00004647-199611000-00033 8898711

[B21] McDannold N Vykhodtseva N Jolesz FA Hynynen K MRI investigation of the threshold for thermally induced blood-brain barrier disruption and brain tissue damage in the rabbit brain Magn Reson Med 2004 51 913 923 10.1002/mrm.20060 15122673

[B22] Vernooij MW Ikram MA Tanghe HL Vincent AJ Hofman A Krestin GP Niessen WJ Breteler MM van der LA Incidental findings on brain MRI in the general population N Engl J Med 2007 357 1821 1828 10.1056/NEJMoa070972 17978290

[B23] Nilsson P Sandberg-Wollheim M Norrving B Larsson EM The role of MRI of the brain and spinal cord, and CSF examination for the diagnosis of primary progressive multiple sclerosis Eur J Neurol 2007 14 1292 1295 10.1111/j.1468-1331.2007.01932.x 17764461

[B24] De Vita E Thomas DL Roberts S Parkes HG Turner R Kinchesh P Shmueli K Yousry TA Ordidge RJ High resolution MRI of the brain at 4.7 Tesla using fast spin echo imaging Br J Radiol 2003 76 631 637 10.1259/bjr/69317841 14500278

[B25] Bernstein MA Huston J Lin C Gibbs GF Felmlee JP High-resolution intracranial and cervical MRA at 3.0T: technical considerations and initial experience Magn Reson Med 2001 46 955 962 10.1002/mrm.1282 11675648

[B26] Pfeuffer J Adriany G Shmuel A Yacoub E Moortele PF Van De Hu X Ugurbil K Perfusion-based high-resolution functional imaging in the human brain at 7 Tesla Magn Reson Med 2002 47 903 911 10.1002/mrm.10154 11979569

[B27] Glover PM Bowtell RW Brown GD Mansfield P A microscope slide probe for high resolution imaging at 11.7 Tesla Magn Reson Med 1994 31 423 428 10.1002/mrm.1910310411 8208118

[B28] Gossl M Malyar NM Rosol M Beighley PE Ritman EL Impact of coronary vasa vasorum functional structure on coronary vessel wall perfusion distribution Am J Physiol Heart Circ Physiol 2003 285 H2019 H2026 12855425 10.1152/ajpheart.00399.2003

[B29] Gossl M Rosol M Malyar NM Fitzpatrick LA Beighley PE Zamir M Ritman EL Functional anatomy and hemodynamic characteristics of vasa vasorum in the walls of porcine coronary arteries Anat Rec A Discov Mol Cell Evol Biol 2003 272 526 537 10.1002/ar.a.10060 12740947

[B30] Gossl M Zamir M Ritman EL Vasa vasorum growth in the coronary arteries of newborn pigs Anat Embryol (Berl) 2004 208 351 37 15309629 10.1007/s00429-004-0400-7

[B31] Gossl M Beighley PE Malyar NM Ritman EL Role of vasa vasorum in transendothelial solute transport in the coronary vessel wall: a study with cryostatic micro-CT Am J Physiol Heart Circ Physiol 2004 287 H2346 H2351 10.1152/ajpheart.00066.2004 15178545

